# A Double-Blind Trial to Test if NAD+ Improves Cognition

**DOI:** 10.1093/geroni/igaf122.3517

**Published:** 2025-12-31

**Authors:** Karina Tavares, Cindy Tsotsoros

**Affiliations:** University of Rhode Island, Providence, Rhode Island, United States; University of Rhode Island, Providence, Rhode Island, United States

## Abstract

Pharmaceutical companies market nicotinamide adenine dinucleotide (NAD+) as a supplement that helps support cognitive functioning in aging adults, selling it as a potential shield against age-related decline and Alzheimer’s disease. Although clinical trials are emerging, current research on its efficacy remains limited. This double-blind clinical trial aimed to determine if individuals who consumed oral NAD+ would show greater improvement on cognitive test scores than those taking a placebo. Thirty-seven women residing in New England who identified as Latina (17 NAD+, 18 placebo; 10 English speakers, 25 Spanish speakers) completed the NIH Toolbox Cognition Battery at baseline and after 28 days of daily supplementation. Measures included the Flanker Inhibitory Control and Attention, Dimensional Change Card Sort, List Sorting Working Memory, Pattern Comparison Processing Speed, and Picture Sequence Memory tasks. These tests assess executive function, working memory, and processing speed. Fully adjusted T-scores were analyzed. Paired-samples t-tests revealed significant pre-post improvements across all measures (p < .001–.079) with medium-to-large effect sizes (NAD+ Cohen’s d = 5.623–11.986; placebo d = 5.140–10.453). ANCOVAs adjusted for baseline revealed no significant between-group differences. English speakers outperformed Spanish speakers, though language did not moderate treatment effects. Findings suggest NAD+ supplementation did not improve cognition more than the placebo. However, medium effect sizes suggest larger studies may reveal treatment effects. This work emphasizes the importance of including women and underrepresented populations in cognitive aging research, and highlights the need for methodologically sound trials to determine whether supplements produce the cognitive benefits they claim.

